# The dark side of mental toughness: a meta-analysis of the relationship between the dark triad traits and mental toughness

**DOI:** 10.3389/fpsyg.2024.1403530

**Published:** 2024-07-18

**Authors:** Taihe Liang, Xianfei Wang, Sanfan Ng, Xuefeng Xu, Ziheng Ning

**Affiliations:** ^1^Institute of Physical Education, Hainan Normal University, Hainan, China; ^2^Faculty of Health Sciences and Sports, Macao Polytechnic University, Macao, China; ^3^Center for Cognition and Brain Disorders, Hangzhou Normal University, Hangzhou, China

**Keywords:** dark triad, narcissism, machiavellanism, psychopathy, mental toughness, meta - analysis

## Abstract

**Introduction:**

This meta-analysis investigates the relationships between the Dark Triad personality traits (narcissism, Machiavellianism, and psychopathy) and mental toughness. Previous research has shown mixed results regarding the influence of these traits on mental toughness. The objective of this meta-analysis is to synthesize existing literature and provide a comprehensive understanding of how Dark Triad traits correlate with mental toughness.

**Methods:**

A comprehensive literature search was conducted in 7 databases, Data were extracted by correlation and analyzed using a random-effects model.

**Results:**

The results yielding 27 effect sizes with a total of 12,378 participants, revealed a significant moderate positive association between narcissism and mental toughness (*r* = 0.327, *p* < 0.001), suggesting that individuals with higher levels of narcissism tend to exhibit greater mental toughness. However, no significant associations were found between Machiavellianism (*r* = 0.023, *p* = 0.719) or psychopathy (*r* = −0.022, *p* = 0.625) and mental toughness.

**Discussion:**

The findings contribute to a more nuanced understanding of the Dark Triad traits and their differential associations with adaptive psychological constructs, highlighting the unique role of narcissism in mental toughness. This meta-analysis provides valuable insights for future research and practical applications in fostering adaptive aspects of narcissism while mitigating its potential maladaptive consequences.

## 1 Introduction

Dark Triad traits, comprising narcissism, Machiavellianism, and psychopathy, are often regarded as undesirable personality (Paulhus and Williams, 2002a). However, an increasing number of studies have begun to investigate the potential “bright” sides of these traits (Papageorgiou et al., 2019b; Neumann et al., 2020; Kun et al., 2021; Lebuda et al., 2021; Zheng and MacCann, 2023). Mental toughness, defined as an individual’s capacity to maintain commitment, confront challenges, and achieve goals in the face of adversity or stressful circumstances, is commonly observed among elite athletes (Jones et al., 2002). Although there is some foundational research on the relationship between Dark Triad traits and mental toughness, there has been no systematic review or meta-analysis in this field to date. Therefore, this study aims to assess the relationship between the two through systematic review and meta-analysis, seeking to address this gap in the literature.

### 1.1 Dark triad

The Dark Triad, encompassing narcissism, Machiavellianism, and psychopathy, has garnered considerable attention in psychological research due to its strong association with socially aversive behaviors (Paulhus and Williams, 2002a). These traits share several core elements including callousness, disagreeableness, interpersonal exploitation, and a propensity for socially aversive actions (Jakobwitz and Egan, 2006a; Jones and Paulhus, 2010; Jones and Figueredo, 2013). Despite the interconnectedness of these dimensions, each trait exhibits distinctive features and behavioral patterns. Individuals scoring high in Narcissism is primarily characterized by an inflated sense of importance, entitlement, dominance, and superiority (Campbell and Baumeister, 2006; Campbell and Miller, 2011). Individuals scoring high in Machiavellianism display a cynical worldview, a lack of principled living, and believe that manipulating others is key to success (Geis and Moon, 1981; Jones and Paulhus, 2010). Individuals scoring high in Psychopathy is typically marked by emotional detachment, ruthlessness, and irresponsibility (Cooke and Michie, 2001).

Currently, Dark Triad research is mostly focused on two concise measures. One of the references is the Dirty Dozen (DTDD; Jonason and Webster, 2010). The Dirty Dozen focuses on brevity using only four items for each member of the triumvirate. While certain researchers have found it beneficial, others have expressed criticism toward it (Furnham et al., 2013). Another widely used metric is the Short Dark Triad (SD3; Jones and Paulhus, 2014). The initial study by the SD3 authors included five experiments, which encompassed informant validation of all three subscales. The SD3 demonstrates better predictive power compared to the Dirty Dozen (Furnham et al., 2013).

### 1.2 Narcissism

The concept of narcissism derives from the Greek myth of Narcissus, an individual who fell in love with his own reflection. This mythological origin lays the foundation for the modern psychological understanding of narcissism, which is extensively examined both as a pathological facet of narcissistic personality disorder and as a broader personality trait (Campbell and Miller, 2011). Clinical narcissism has been a subject of significant scholarly interest, where individuals often exhibit characteristics such as exaggeration, dominance, and a marked sense of superiority (Campbell et al., 2002; Campbell and Baumeister, 2006). Subclinical narcissism is recognized as a multidimensional construct, primarily divided into grandiose narcissism and vulnerable narcissism (Wink, 1991; Miller et al., 2011). Individuals with high levels of grandiose narcissism often show boastfulness, lack of humility, and tendencies toward interpersonal domination. Conversely, those characterized by vulnerable narcissism typically experience intense negative emotions, distrust, self-centeredness, and a profound need for attention and recognition (Back et al., 2013).

In the realm of research, the Narcissistic Personality Inventory (NPI) remains a widely used tool for assessing narcissism. This inventory has been pivotal in the study of Dark Triad traits, incorporating both the comprehensive original 40-item NPI (Raskin and Hall, 1979) and its streamlined version, the NPI-16 (Ames et al., 2006). Notably, the NPI’s measurement of grandiose narcissism correlates strongly with other dark personality traits, underlining the intricate connections within the Dark Triad (Furnham et al., 2013).

### 1.3 Machiavellianism

Machiavellianism derives its name from Niccolò Machiavelli, a 16th-century Italian writer whose seminal work, “The Prince,” left an indelible mark on political thought. Unlike pathological personality disorders, Machiavellianism encapsulates a distinct behavioral style in political contexts, characterized by suave social charm, high manipulativeness, and a blatant disregard for conventional morality (Christie, 1970a). This trait typically manifests through strategic exploitation of others using charm and seduction to achieve personal gain (Turner and Martinez, 1977; Jonason and Webster, 2012).

The measurement of Machiavellianism has been historically anchored by the Mach-IV scale, developed by Christie (1970b), which remains a prominent instrument in psychological research. This scale is also integrated into newer assessments like the Short Dark Triad (Jones and Paulhus, 2014), which captures broader dimensions of the Dark Triad traits, including Machiavellianism.

Further advancements in understanding this trait have been highlighted in recent studies, such as those by Truhan et al. (2021), which have refined the characterization of Machiavellianism. These studies delineate it into specific components: manipulation, a pervasive lack of morals and ethics, detachment, and cynicism, enhancing our comprehension of its multifaceted nature.

### 1.4 Psychopathy

Psychopathy is distinguished by traits such as superficial charm, exploitativeness, irresponsibility, and impulsivity (Hart et al., 1992). It is frequently misconstrued with antisocial personality disorder; however, while the latter is characterized by behavioral manifestations, psychopathy is rooted in distinct personality traits (Berg et al., 2013). Psychopaths are known for manipulating and exploiting interpersonal relationships to serve their own ends (Hare, 1985, 1999), engaging in impulsive and often destructive behaviors without regard for the consequences to themselves or others.

At a subclinical level, psychopathy is considered the most malignant aspect of the Dark Triad, associated with arrogant and deceitful interpersonal styles, as well as impulsive and irresponsible risk-taking behaviors (Crysel et al., 2013; Jonason and Ferrell, 2016; Malesza and Ostaszewski, 2016). These individuals typically exhibit a short-term goal orientation and a diminished need for belonging, frequently employing both reactive and instrumental aggression to achieve their goals (Schoenleber et al., 2011), and are known for their ruthless strategies to accomplish objectives (Jonason and Webster, 2012; Jones and Figueredo, 2013; Kranefeld, 2023).

The Triarchic Model of Psychopathy outlines three distinct phenotypic constructs of psychopathy: disinhibition, which indicates a general predisposition toward impulse control issues; boldness, characterized by social dominance, emotional resilience, and venturesomeness; and meanness, defined by aggressive resource-seeking without regard for others (Patrick et al., 2009; Sleep et al., 2019). In psychometric assessments, the Self-Centered Impulsivity scale of the Psychopathic Personality Inventory (PPI) is notably correlated with dark personality traits (Lilienfeld and Andrews, 1996), while the Fearless Dominance factor is associated primarily with adaptive correlates (Lynam and Miller, 2012).

### 1.5 Mental toughness

Mental toughness was originally conceptualized in the context of athletic research, identified as a set of traits closely linked to exceptional sporting performance (Jones et al., 2002). It is associated with numerous positive outcomes, such as enhanced adaptability, resolute goal pursuit, and robust coping strategies (Jones et al., 2002; Perry et al., 2021). Generally, mental toughness is perceived as a personality construct that enables individuals to thrive in stressful environments, persist in their goals, and maintain confidence in adversity. Scholars hold varying views on the composition of mental toughness; some advocate for its multidimensionality (Clough et al., 2002; Jones et al., 2002; Perry et al., 2021), while others support a unidimensional model (Gucciardi et al., 2015a; Gucciardi, 2018). The most widely accepted definition today is the four-dimensional model proposed by Clough et al. (2002), comprising: (1) Control: the individual’s tendency to believe they can influence their life and emotions; (2) Commitment: the persistence in pursuing goals despite difficulties; (3) Challenge: viewing potential threats as opportunities for personal growth; (4) Confidence: maintaining a belief in one’s worth and ability to advance socially, even in the face of setbacks.

Various scales have been developed to measure mental toughness, including the Mental Toughness Questionnaire 48 (MTQ48; Clough et al., 2002) and its abridged version, the MTQ18 and MTQ10 (Clough et al., 2002; Dagnall et al., 2019), the Sports Mental Toughness Questionnaire (SMTQ; Sheard et al., 2009), the Cricket Mental Toughness Inventory (CMTI; Gucciardi and Gordon, 2009), the Australian Football Mental Toughness Inventory (Gucciardi et al., 2009), and the Mental Toughness Index (MTI; Gucciardi et al., 2015b). Among these, the MTQ, based on Clough et al.’s 4C model, is the most extensively studied and used tool for measuring mental toughness (Birch et al., 2017). Despite some controversy over its applicability in certain domains (Gucciardi, 2018), it is considered the “gold standard” for measuring mental toughness to date (Perry et al., 2021). In recent years, mental toughness has also garnered widespread attention in the fields of workplace and education (Lin et al., 2017).

### 1.6 Dark triad and mental toughness

While traditional views often emphasize the maladaptive and pathological aspects of the Dark Triad (Rauthmann and Kolar, 2012; Jones and Paulhus, 2014; Paulhus, 2020), recent research has begun to explore the connections between these traits and adaptive psychological characteristics (Wallace et al., 2009; Zeigler-Hill et al., 2019; Lebuda et al., 2021). Researchers have gradually identified overlaps between the Dark Triad and the concept of mental toughness. Both individuals with high narcissism and mental toughness exhibit significant self-confidence. Not only is it positively correlated with narcissism (Papageorgiou et al., 2018a,b), but it is also a central element in mental toughness (Bull et al., 2005; Thelwell et al., 2005; Gucciardi, 2014). Additionally, grandiose narcissism’s negative correlation with stress may suggests that narcissists can overcome adversity or unfavorable environments at any cost to achieve their goals, indicating a potential positive correlation with mental toughness (Papageorgiou et al., 2019a,b).

Individuals with Machiavellian tendencies emphasize short-term gains and are prone to achieve ends by any means necessary (Jonason et al., 2010; Birkás et al., 2015; Kilduff and Galinsky, 2017; Blickle et al., 2020). However, their poor self-control may make it difficult for them to persist in the face of adversity (Jonason and Tost, 2010; Malesza and Ostaszewski, 2016), opting instead to manipulate others or seek alternative strategies rather than facing challenges directly (Geis and Moon, 1981; Wilson et al., 1996), likely resulting in a negative correlation with mental toughness.

Psychopathic individuals may exhibit impulsive and irresponsible behavior under stress (Jonason et al., 2015), lack the necessary self-discipline and are associated with dysfunctional impulsivity (Crysel et al., 2013; Jonason and Ferrell, 2016; Malesza and Ostaszewski, 2016), indicating difficulty in resisting immediate rewards in pursuit of greater delayed gratification (Ainslie, 1975; Crysel et al., 2013; Baughman et al., 2016). This pursuit of immediate gratification can impair decision-making processes (Crone et al., 2003) and lead to reckless and self-destructive behaviors (Hemphill et al., 1998; Hare, 1999). Therefore, psychopathy might be negatively correlated with mental toughness.

Beyond overlapping dimensions between the two variables, other theoretical frameworks also lend insights into the relationship between the Dark Triad and mental toughness. First, the Dark Triad and mental toughness may share a link due to their association with the Big Five personality traits. The most consistent correlations of Dark Triad traits are negatively with agreeableness and conscientiousness (Paulhus and Williams, 2002b; Lee and Ashton, 2005; Jakobwitz and Egan, 2006b; Paulhus, 2014). In the HEXACO model, Honesty–Humility is nearly antithetical to the Dark Triad (Lee and Ashton, 2005, 2014; Lee et al., 2013). Given the Dark Triad’s poor interpersonal relationships, evasion of responsibility, and habitual lying (Paulhus, 2014), this is not surprising. Conversely, some studies find mental toughness positively correlates with agreeableness and conscientiousness (Veselka et al., 2009; Yankov et al., 2019; Rodrigues et al., 2023). Individuals high in mental toughness can maintain a positive and optimistic commitment under adverse conditions, reflecting both agreeableness and conscientiousness. Also, the commitment dimension of mental toughness highly overlaps with Honesty–Humility (Clough et al., 2002; Ashton et al., 2004). Thus, the inverse relationship with the Big Five and HEXACO may imply a negative correlation between the Dark Triad and mental toughness.

Secondly, evolutionary psychological survival strategies might also ground the relationship between the Dark Triad and mental toughness. Life history strategies range from delayed to immediate gratification (Figueredo et al., 2005, 2014). Individuals with Dark Triad traits tending toward immediate gratification characterized by poor self-control, frequent short-term mating, selfishness, and other antisocial behaviors. Each member of the Dark Triad exploits others in unique social contexts, and their callous exploitation has promoted reproductive success. Although Dark Triad generally tend toward immediate gratification (Jonason and Tost, 2010), narcissism exhibits the least tendency in this regard (Baughman et al., 2016). Compared to the other traits, grandiose exhibitionism facets of narcissism are associated with a slow life strategy (Jonason et al., 2010). Therefore, highly narcissistic individuals are more likely to exhibit mental toughness.

Thirdly, the dual-personality framework and differing domain performances might also provide a basis for the potential correlation between the Dark Triad and mental toughness (Leary, 2004). The Dark Triad are characterized by high agency and low communion, meaning, despite generally being unpopular, they can still perform excellently (Hogan et al., 2007). Mental toughness may support individuals with the Dark Triad to endure the stress of unpopularity and relentlessly pursue their goals in unfavorable situations. For instance, in the workplace, the Dark Triad may help individuals become leaders. Narcissism is considered a typical trait of leaders (Rosenthal and Pittinsky, 2006; Brunell et al., 2008). In leadership positions, Individuals with high levels of narcissism are perceived as more charismatic (Braun, 2017; Den Hartog et al., 2020). Some researchers now focus on so-called successful narcissists (Chatterjee and Hambrick, 2007; Chatterjee and Pollock, 2017; Zeigler-Hill et al., 2019). Moreover, when high levels of Dark Triad traits are combined with other factors, such as intelligence, physical attractiveness, they generally aid in attaining leadership positions (Furnham, 2016). Given that the impact of mental toughness in enterprises has been widely recognized (Ruparel, 2020; Lee and Kim, 2023; Ruparel et al., 2023; Suharti and Kurniawati, 2023), mental toughness might also be one of the underlying factors. In education, mental toughness helps students cope with academic pressure and achieve academic success. For instance, narcissism might manifest as academic confidence and the pursuit of excellence. Research suggests that the relationship between narcissism and MT may be one of the non-cognitive mechanisms leading to individual performance differences in school (Papageorgiou et al., 2019b). In contrast, Machiavellianism and psychopathy are more likely to “find alternative paths.” The most obvious examples are cheating and plagiarism in papers. While psychopathy is the only independent predictor of exam cheating (Nathanson et al., 2006), Machiavellianism can also predict paper plagiarism (Williams et al., 2010). In sports, mental toughness is a key factor in maintaining high performance under pressure and challenges for athletes. However, those with darker personalities continually may face the temptation to cut corners, such as using performance-enhancing drugs (Nicholls et al., 2017) and cheating (Potgieter, 2013). Interestingly, narcissism again distinguishes itself from the other dimensions. Athletes with high levels of narcissism do not tend to use unethical means to achieve victory because they have sufficient confidence in their abilities. They are more likely to overtraining, even if it makes them more prone to injuries (Yates, 1996; Tibbert, 2013).

### 1.7 The present study

Despite the insights provided by previous studies, our understanding of the relationships between the Dark Triad and mental toughness remains incomplete. First, previous findings have yielded inconsistent results. Discrepancies exist concerning the relationship between the Dark Triad and mental toughness, evident in the varied directions and magnitudes of their impact. For instance, some researchers have suggested that individuals with high levels of narcissism are more inclined to exhibit mental toughness (Papageorgiou et al., 2018a, 2023), while others have reported no effect or even a negative impact of narcissism on mental toughness (Ryerson, 2018; Milošević et al., 2022).

Further, there is a lack of consensus on which specific personality construct within the Dark Triad has greater explanatory power for mental toughness. For example, certain studies have found that narcissism exhibits the highest correlation with mental toughness, while others contend that Machiavellianism demonstrates the strongest correlation (Sabouri et al., 2016; Papageorgiou et al., 2019b).

Moreover, previous research has primarily focused on discrete contextual factors that may interact with personality constructs to impact mental toughness. However, personality expression is also influenced by domains, such as sport, education, and workplace (Lin et al., 2017; Szabó et al., 2022). To date, few studies have placed domain differences at the forefront of their designs.

To address these research gaps, this study aims to conduct a comprehensive systematic review of the relationship between narcissism, Machiavellianism and psychopathy and mental toughness by meta-analytically examining the impact of the Dark Triad. In this process, we will also assess the relative importance of each Dark Triad trait in predicting and explaining mental toughness, determining which Dark Triad construct has the strongest explanatory power.

Based on previous studies and the Dark Dyad theory (Papageorgiou et al., 2019b; Rogoza and Cieciuch, 2020), which posits that narcissism is more likely to be positively correlated with adaptive psychological traits, we hypothesize that:

*H1:* Narcissism will be positively associated with mental toughness.

In contrast, Machiavellianism and psychopathy, as components of the dark dyad, are more likely to be negatively correlated with adaptive psychological traits. We hypothesize that:

*H2:* Machiavellianism will be negatively associated with mental toughness.

*H3:* Psychopathy will be negatively associated with mental toughness.

Furthermore, previous research has highlighted the potential influence of domains such as sports, education, and the workplace. Therefore, this study will examine domains as possible moderators of the relationship between the Dark Triad and mental toughness to account for the inconsistent findings observed in previous studies.

Overall, this research makes three main contributions. First, to the best of our knowledge, this study is the first to use meta-analysis to clarify the relationships between the Dark Triad and mental toughness. Second, by comparing the frameworks of the Dark Triad, we present a more comprehensive understanding of these personality constructs and demonstrate whether narcissism has a brighter side compared to Machiavellianism and psychopathy in relation to mental toughness. Finally, we contribute to the literature by exploring the moderating role of domains in the relationships among the Dark Triad traits and mental toughness.

## 2 Methods

### 2.1 Literature search

To ensure comprehensive coverage, a literature search was executed across seven electronic databases: ScienceDirect, ProQuest, PsycINFO, Web of Science, PubMed, CNKI, and Google Scholar. The search utilized a combination of keywords tailored to each database’s specific syntax requirements. Keywords included permutations of (“Dark Triad” OR Narcissis* OR Machiavellian* OR Psychopathy OR Psychopathic) AND (Mental Toughness OR mentally tough). These terms targeted titles, abstracts, and keywords of articles. The search was conducted on February 16, 2024, with no date limitations imposed. Additionally, a manual search of reference lists from relevant articles was conducted to identify further studies.

### 2.2 Inclusion criteria

Inclusion in the meta-analysis required studies to: (1) empirically examine the relationship between at least one Dark Triad trait and mental toughness; (2) utilize validated measures for both constructs as presented in the introduction; (3) provide sufficient data (e.g., Pearson’s correlation coefficients, sample sizes) for effect size extraction; and (4) be published in English.

### 2.3 Study selection and data extraction

Following PRISMA guidelines (Moher et al., 2009), the selection process began by removing duplicates, followed by a preliminary screening of titles and abstracts by two independent researchers. Discrepancies were resolved via discussion until a consensus was reached. Potentially eligible articles underwent full-text review for final inclusion.

**Figure 1:**
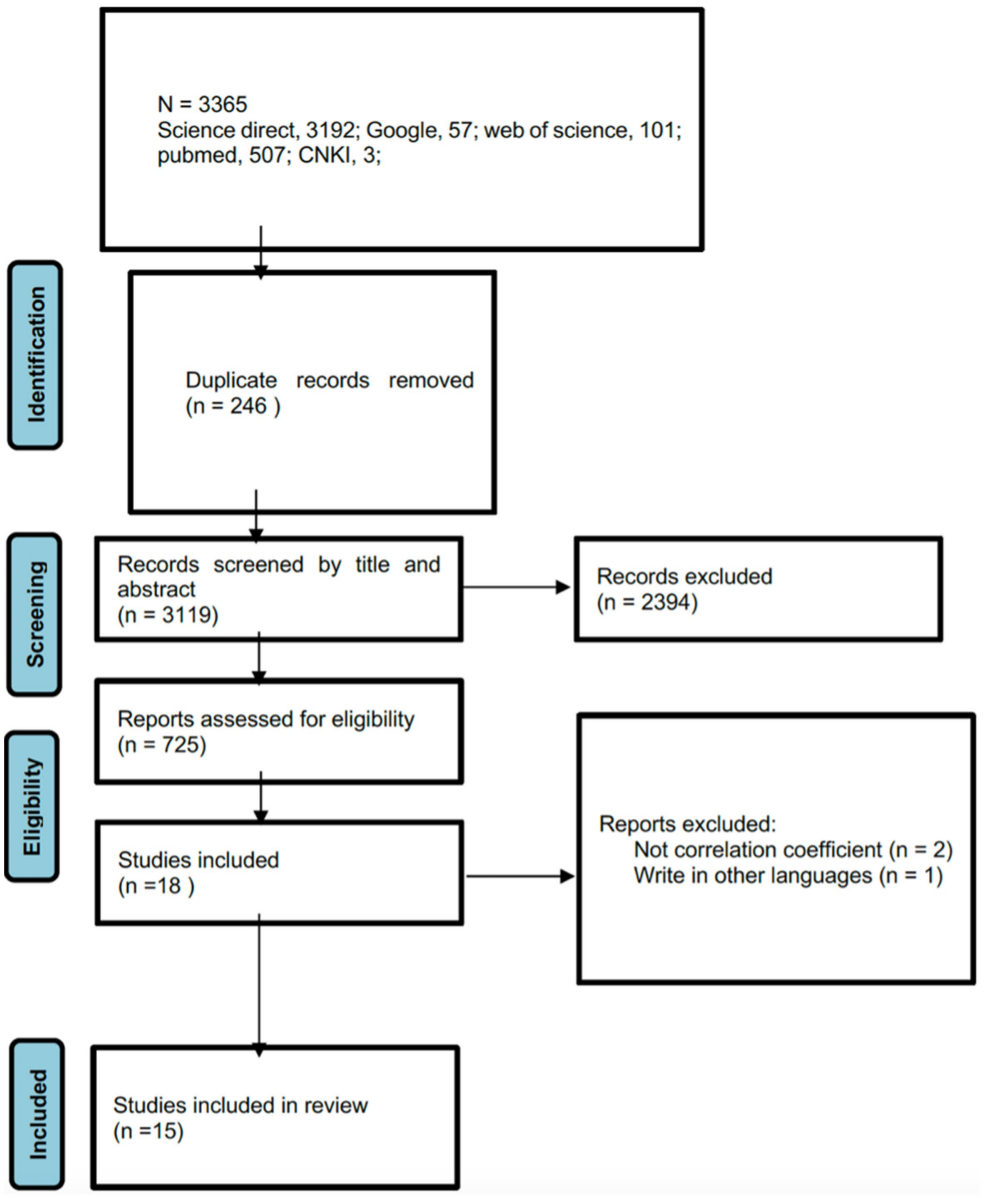
PRISMA flow diagram of search outcomes for the relationship between the Dark Triad and mental toughness.

Data extraction was also independently conducted by two researchers using a standardized form, capturing details such as study and sample domains, measures used, and effect sizes. Disagreements were addressed through discussion, with a fifth researcher consulted as necessary. The process and results of the searches and screenings are illustrated in Figure 1.

### 2.4 Statistical analysis

Effect sizes were derived from Pearson’s correlation coefficients between the Dark Triad traits and mental toughness, transformed using Fisher’s z-transformation, and analyzed using a random-effects model. Random-effects model accommodating both within-study and between-study variability, provides conservative and generalizable estimates (Borenstein et al., 2010).

Heterogeneity was evaluated using Cochran’s Q test and the I^2^ statistic, with significant heterogeneity indicated by I^2^ values over 50% (Higgins, 2003). Subgroup analyses were conducted to explore potential moderators like domains and measurement tools in cases of significant heterogeneity. Publication bias was assessed using funnel plots and Egger’s regression test (Egger et al., 1997), with adjustments made using the trim-and-fill method (Duval and Tweedie, 2000) if necessary.

All statistical analyses were conducted using Comprehensive Meta-Analysis software, with significance set at a two-tailed *p*-value of less than 0.05. This study has been pre-registered in Prospero (CRD42023460188).

## 3 Results

An overview of the 15 studies included in this review, encompassing 27 samples and 53 effect sizes (*k* = 15, *N* = 12,378), which is presented in Table 1. Each study employed a cross-sectional design, with sample sizes varying from 92 to 1,238 participants. The studies were conducted across a diverse set of countries, including the United States, Canada, Australia, Italy, Iran, Hungary, Greece, Serbia, Germany, Thailand, and the United Kingdom and so on.

**Table 1:** Included studies.

K	First author and year	n	MT tool	DT tool	N	M	P
1	Papageorgiou et al. (2017)	608	MTQ-48	SD3	0.20	−0.07	−0.08
2	Papageorgiou et al. (2018a)	927	MTQ-10	SD3	0.38		
3	Papageorgiou et al. (2018b)	561	MTQ-10	SD3	0.34		
4	Szabó et al. (2022)	189	Self developed	SD3	0.47	0.00	−0.02
5	Szabó et al. (2022)	134	Self developed	SD3	0.33	−0.03	0.03
6	Szabó et al. (2022)	345	Self developed	SD3	0.34	0.02	0.16
7	Sabouri et al. (2016)	341	MTQ-18	NPI	0.50	0.45	0.20
8	Zamani Sani et al. (2022)	308	SMTQ	SD3	0.21	−0.05	−0.23
9	Zamani Sani et al. (2022)	152	SMTQ	SDS3	0.24	0.04	0.00
10	Kun et al. (2021)	189	Self developed	SD3	0.47	0.00	−0.02
11	Denovan et al. (2021)	252	MTQ-10	SD3	0.24	−0.06	0.04
12	Denovan et al. (2021)	154	MTQ-10	SD3	0.36	0.04	−0.04
13	Papageorgiou et al. (2019b)	364	MTQ-48	SD3	0.44		
14	Papageorgiou et al. (2019b)	364	MTQ-48	SD3	0.55		
15	Papageorgiou et al. (2019b)	144	MTQ-48	SD3	0.46		
16	Vaughan et al. (2018)	762	MTQ-48	SD3	0.28	−0.30	−0.26
17	Manley et al. (2019)	297	MTI	NARQ	0.15		
18	Truhan et al. (2022)	611	MTQ-10	SD3	0.41		
19	Truhan et al. (2022)	1,235	MTQ-10	SD3	0.28		
20	Papageorgiou et al. (2023)	616	MTQ-10	SD3	0.40		
21	Papageorgiou et al. (2023)	1,238	MTQ-10	SD3	0.28		
22	Papageorgiou et al. (2023)	428	MTQ-10	SD3	0.27		
23	Papageorgiou et al. (2023)	1,100	MTQ-10	SD3	0.33		
24	Papageorgiou et al. (2023)	267	MTQ-10	SD3	0.39		
25	Kinrade et al. (2022)	444	MTQ-48	NPI	0.33		
26	Milošević et al. (2022)	92	MTI	DDTD	−0.04	0.38	0.00
27	Ryerson (2018)	256	MTQ-18	DDTD	−0.06	−0.11	−0.03

A significant majority of the samples (20 out of 27) had a predominance of female participants. The domains of the samples were varied: eleven studies targeted university students, five focused on athletes, and three involved university employees. It is worth mentioning that four of the samples involved subjects who were both students and athletes. The measurement of Dark Triad traits predominantly utilized brief instruments: 20 samples employed the Short Dark Triad scale (SD3), three used the Dirty Dozen Measure (DDTD), and three assessed only narcissism, with two of these using the Narcissistic Admiration and Rivalry Questionnaire (NARQ) and one utilizing the Narcissistic Personality Inventory (NPI).

In terms of assessing mental toughness, a variety of instruments were used. The Mental Toughness Questionnaire (MTQ) was the most commonly employed, used in 18 samples. Additionally, two samples utilized the Mental Toughness Index (MTI), two employed the Sports Mental Toughness Questionnaire (SMTQ), and four used custom-developed measures.

### 3.1 Narcissism and mental toughness

The correlation between narcissism and mental toughness was robustly investigated across 27 samples from 15 distinct studies, encompassing a diverse range of sample sizes from 92 to 1,238 participants. Consistently, all measured effect sizes indicated a significant positive correlation between narcissism and mental toughness. The instruments used for assessing narcissism varied, with 20 samples utilizing the Short Dark Triad scale (SD3), two using the Narcissistic Personality Inventory (NPI), four employing the Dirty Dozen (DDTD), and one opting for the Narcissistic Admiration and Rivalry Questionnaire (NARQ). It is worth mentioning that the two non-significant effect sizes were both from the DDTD scale. While one study assessed both grandiose narcissism and vulnerable narcissism, we only extracted the correlation coefficient between grandiose narcissism and mental toughness, as narcissism present in individuals with the Dark Triad is characterized by grandiose narcissism. These findings uniformly support Hypothesis 1, confirming a stable positive association between narcissism and mental toughness across different study contexts and measurement tools.

### 3.2 Machiavellianism and mental toughness

This analysis involved 13 samples from nine studies, with sample sizes ranging between 92 and 762. The relationship between Machiavellianism and mental toughness showed mixed results: while the majority of samples reported no significant correlation, three studies did identify significant correlations, though they were inconsistent. Specifically, one study using the MACH-IV to measure Machiavellianism found a positive correlation with mental toughness, while two other studies, employing the Short Dark Triad (SD3) and the Dirty Dozen (DDTD) scales, found negative correlations. Given these mixed findings, the overall relationship between Machiavellianism and mental toughness remains inconclusive.

### 3.3 Psychopathy and mental toughness

Similarly, 13 samples from nine studies explored the association between psychopathy and mental toughness, with sample sizes ranging from 92 to 762. Of these, five samples indicated significant correlations: two reported positive correlations using the SRP-III and DDTD, while three found negative correlations, two using SD3 and one with DDTD. The other studies did not demonstrate a significant relationship. Thus, although a negative correlation was more frequently observed, the evidence regarding the relationship between psychopathy and mental toughness remains mixed and underscores the complexity of these interactions.

### 3.4 Meta-analysis

**Figure 2:**
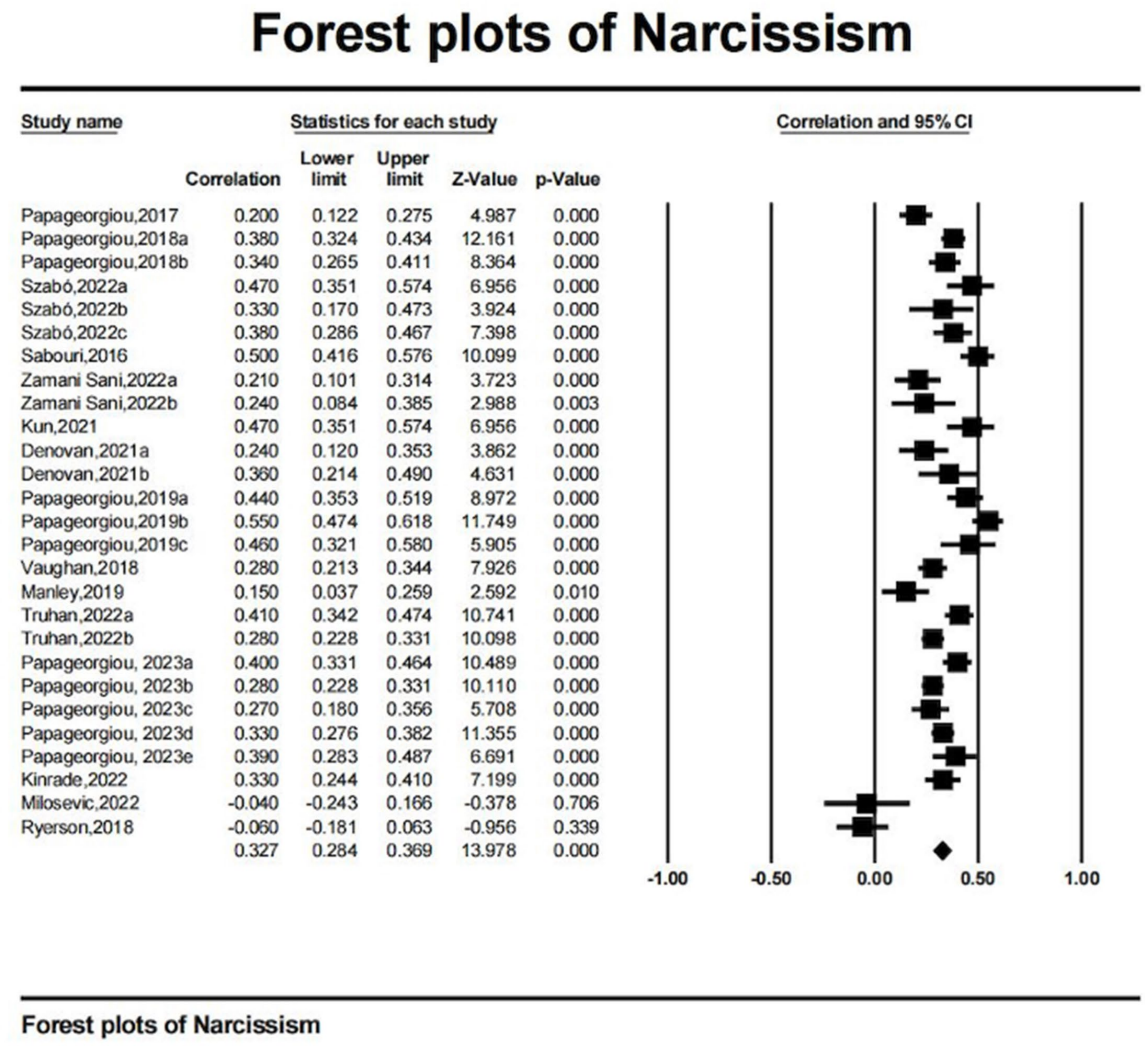
Forest plots of narcissism.

**Figure 3:**
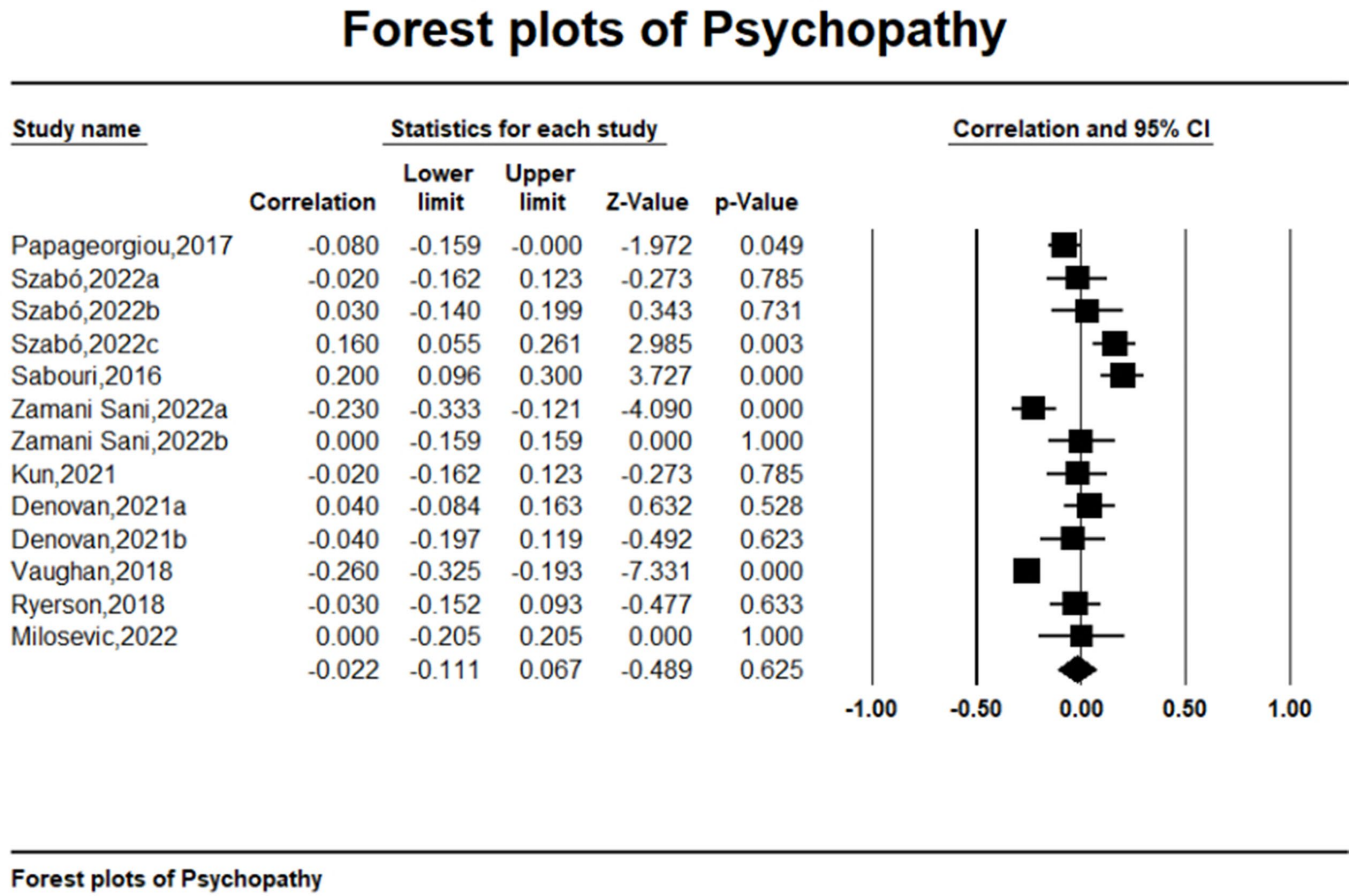
Forest plots of psychopathy.

**Figure 4:**
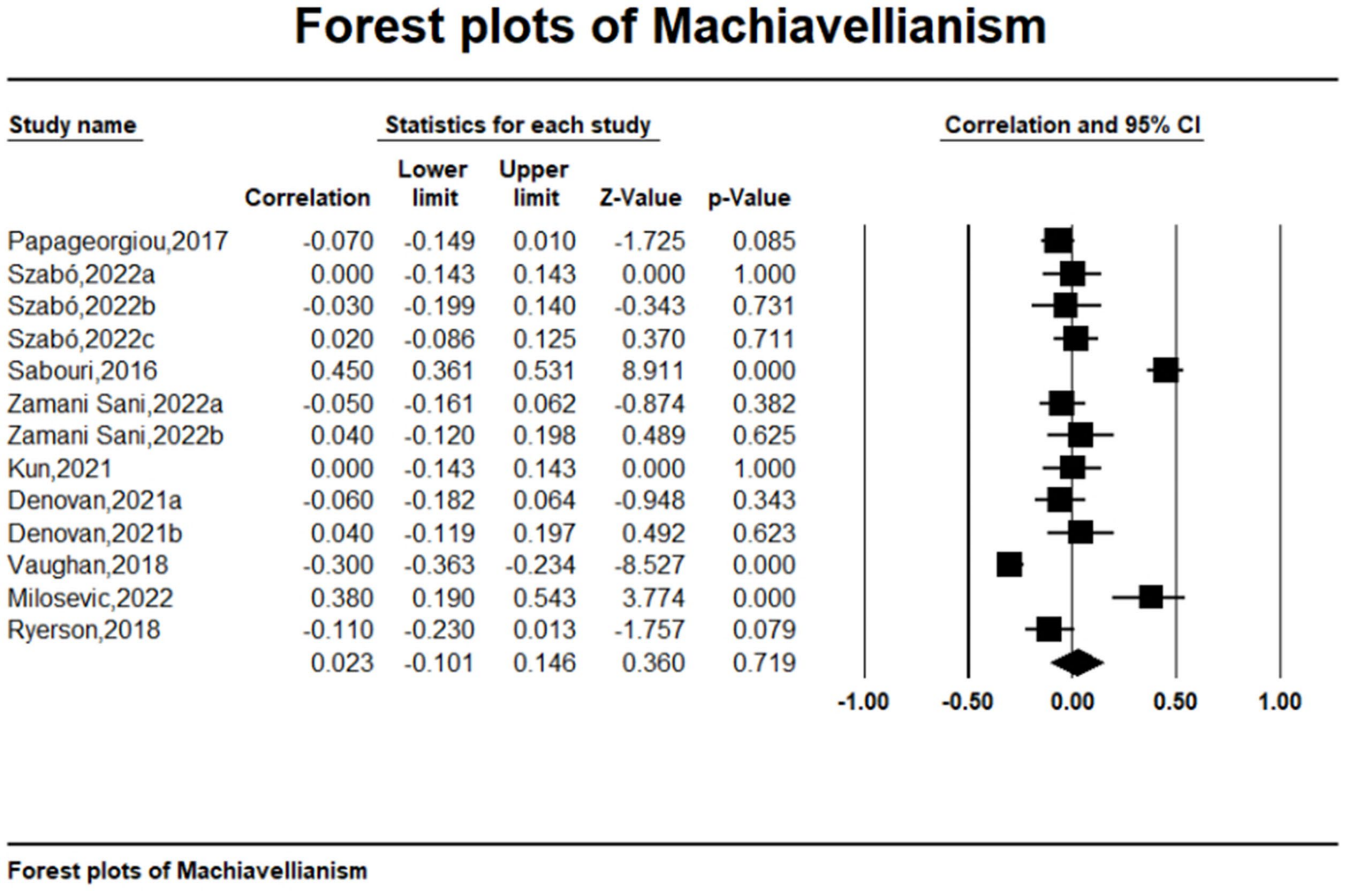
Forests plots of Machiavellianism.

Meta-analytic results are detailed in Table 2 and visually represented in Forest Plot (Figures 2–4). The analysis revealed a significant small to moderate positive correlation between narcissism and mental toughness (*r* = 0.327, *k* = 27, *N* = 12,378; 95% CI = 0.08 to 0.27; *Q* = 173.693; *I^2^* = 85.03%), substantiating Hypothesis 1. Conversely, the overall correlation between Machiavellianism and mental toughness was not significant and exhibited a small effect size (*r* = −0.032, *k* = 13, *N* = 3,782; 95% CI = −0.15 to 0.09; *Q* = 164.93; *I^2^* = 92.73%), providing no support for Hypothesis H2. The relationship between psychopathy and mental toughness also showed no significant correlation (*r* = −0.022, *k* = 13, *N* = 3,782; 95% CI = −0.26 to 0.13; *Q* = 87.03; *I^2^* = 86.21%).

**Table 2:** Overall effect sizes.

	Sample size	Number Studies	Point estimate	Lower limit	Upper limit	Z-value	P-value
N	12,378	27	0.327	0.284	0.369	13.978	0.000
M	3,782	13	0.023	−0.101	0.146	0.360	0.719
P	3,782	13	−0.022	−0.111	0.067	−0.489	0.625

**Figure 5:**
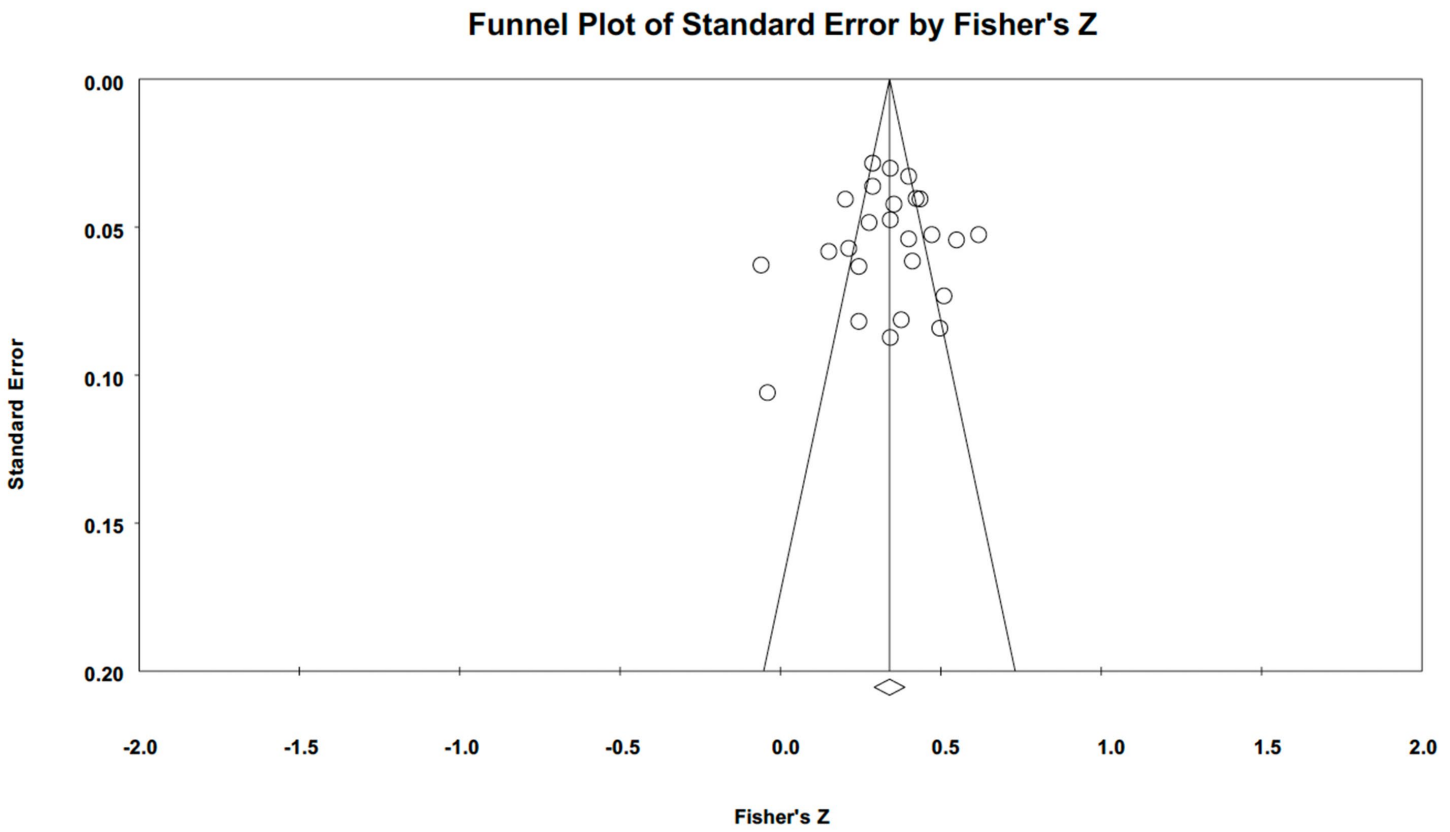
Funnel plot for narcissism.

**Figure 6:**
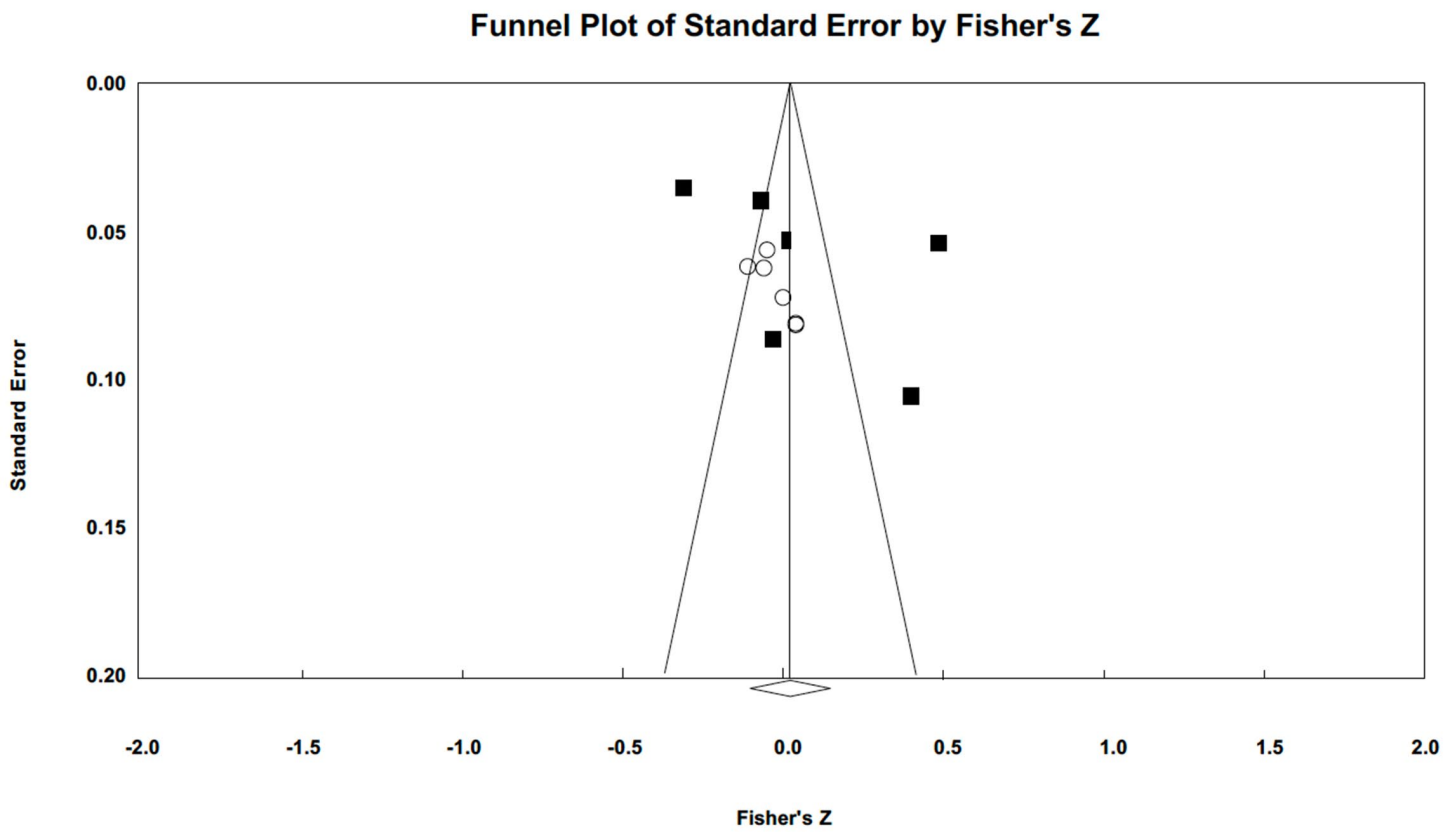
Funnel plot for Machiavellianism.

**Figure 7:**
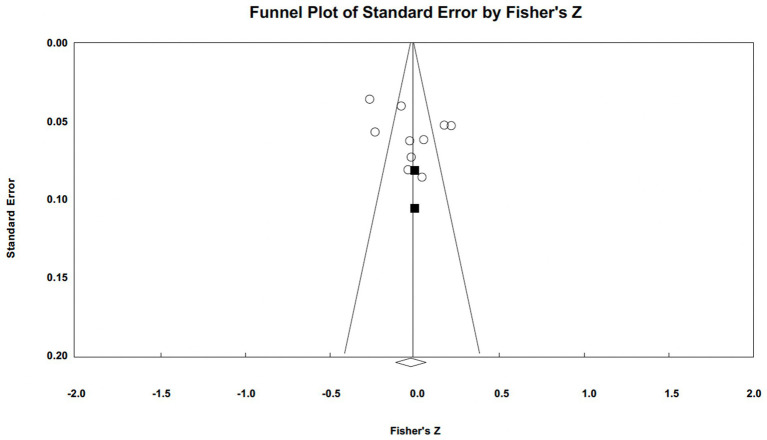
Funnel plot for psychopathy.

**Figure 8:**
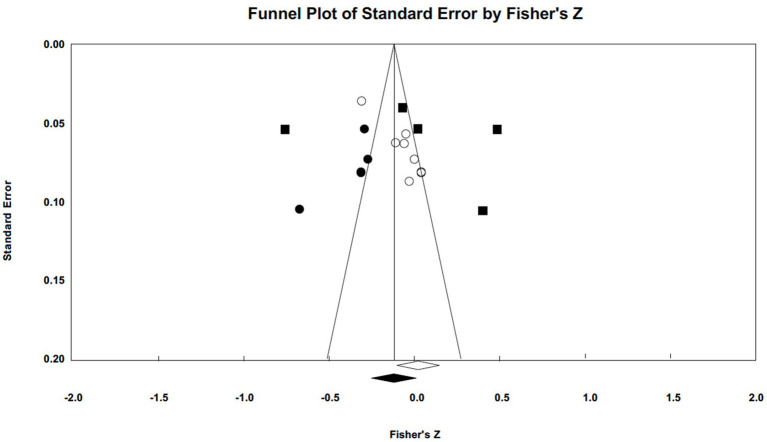
Funnel plot for Machiavellianism (trim and fill).

**Figure 9:**
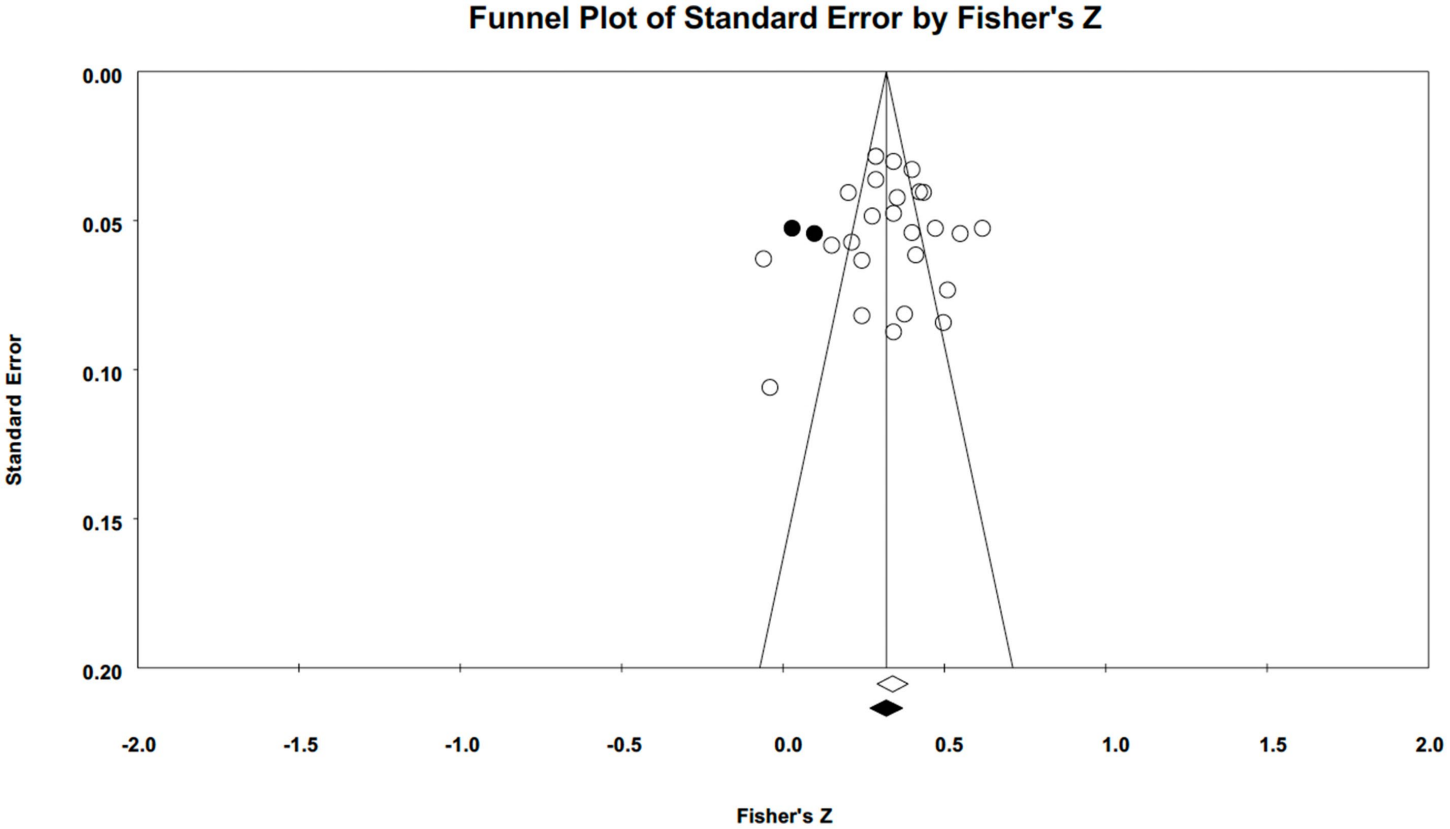
Funnel plot for narcissism (trim and fill).

**Figure 10:**
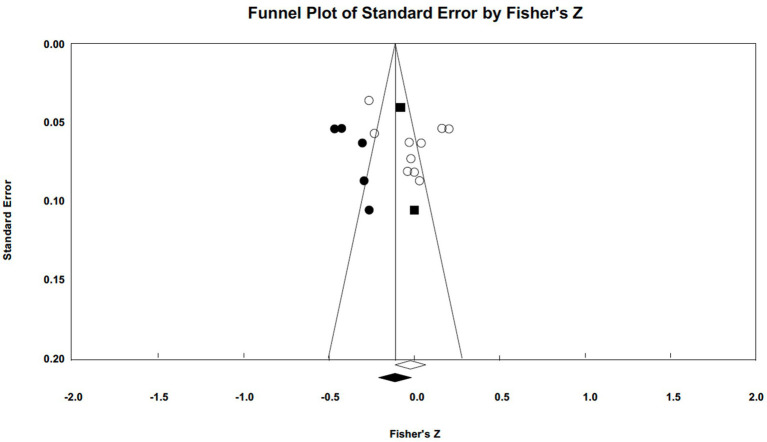
Funnel plot for psychopathy (trim and fill).

Publication bias was assessed using funnel plots and Egger’s tests (illustrated in Figures 5–7 for narcissism, Machiavellianism, and psychopathy, respectively). The funnel plots indicated no publication bias for Machiavellianism (Figure 8). However, the plots for narcissism and psychopathy were asymmetrical, suggesting a potential omission of studies. Employing the trim and fill procedure to adjust for this bias, the association between narcissism and mental toughness remained positive and significant after four adjustment values (*r* = 0.30; 95% CI = 0.29 to 0.34; *Q* = 255.38; *I^2^* = 78.75%), supporting Hypothesis 1 (Figure 9). However, although the association between psychopathy and mental toughness remained negative, it was still non-significant (*r* = −0.11; 95% CI = −0.13 to −0.08), hence not supporting Hypothesis 3 (Figure 10).

Subgroup analyses were conducted to explore potential moderators of the relationship between narcissism and mental toughness. The moderators examined included domains, Dark Triad measurement tools, and mental toughness measurement tools. Across all domains, a positive correlation persisted, though the strength varied significantly. Athletes demonstrated the weakest correlation (*r* = 0.168; 95% CI = 0.048 to 0.283; *p* = 0.006), followed by students (*r* = 0.264; 95% CI = 0.178 to 0.347; *p* < 0.001), and corporate personnel exhibited the strongest correlation (*r* = 0.429; 95% CI = 0.365 to 0.489; *p* < 0.001).

Regarding the measurement tools, the SD3 scale consistently reported higher correlations between narcissism and mental toughness compared to other scales. Interestingly, studies using the Dirty Dozen (DDTD) scale did not find a significant relationship between narcissism and mental toughness. However, due to the limited number of studies utilizing the DDTD scale, its impact remains uncertain (Table 4).

Given the relatively small number of studies, many potential categorical moderators did not have sufficient numbers of effects within each category to conclusively determine the significance of moderation (Table 3).

**Table 3:** Subgroup analyses of domains.

Group	Sample size	Number Studies	Point estimate	Lower limit	Upper limit	*Z*-value	*P*-value
Athletes	1775	5	0.168	0.048	0.283	2.734	0.006
Employees	723	3	0.429	0.365	0.489	11.744	0.000
Internet	7,172	11	0.368	0.309	0.425	11.333	0.000
Students	3,424	11	0.264	0.178	0.347	5.839	0.000

## 4 Discussion

This article represents the first systematic review and meta-analysis exploring the relationship between the Dark Triad traits and mental toughness. Consistent with our first hypothesis, there is a significant moderate positive correlation between narcissism and mental toughness, suggesting that individuals with higher levels of narcissism may associated with greater mental toughness. However, no significant associations were found between Machiavellianism and psychopathy and mental toughness, thus our second and third hypotheses were not supported (Table 4).

**Table 4:** Subgroup analyses of mental toughness tool.

Group	Sample size	Number Studies	Point estimate	Lower limit	Upper limit	*Z*-value	*P*-value
MTI	389	2	0.076	−0.107	0.253	0.815	0.415
MTQ-10	7,389	11	0.335	0.300	0.368	17.703	0.000
MTQ-18	597	2	0.241	−0.338	0.687	0.806	0.421
MTQ-48	2,686	6	0.378	0.267	0.479	6.284	0.000
Self developed	857	4	0.414	0.350	0.474	11.523	0.000
SMTQ	460	2	0.220	0.131	0.305	4.763	0.000

### 4.1 Narcissism and mental toughness

Current research indicates that individuals with high narcissism scores may exhibit higher levels of mental toughness, consistent with our first hypothesis. This finding aligns with previous research, highlighting the adaptive aspects of narcissism (Sedikides et al., 2004; Smith et al., 2016; Sedikides, 2021). One possible reason may stem from the mediating role of self-esteem between narcissism and mental toughness. Baumeister and Vohs (2001) described narcissism as addiction to esteem, which may be a crucial factor in the association between narcissism and mental health aspects. Individuals with high narcissism scores base their self-esteem on self-perceived competence (Tafarodi and Swann, 1995; Tafarodi and Milne, 2002), often possessing an inflated sense of self-worth and a strong desire to be admired. This may drive them to perceive themselves as invincible and capable of maintaining confidence in challenging situations (Rogoza et al., 2018; Grapsas et al., 2020). These reasons align with our proposal that narcissism should positively correlate with mental toughness due to its positive association with self-esteem. Further research indicates that grandiose narcissism in subclinical settings positively correlates with self-esteem. It is worth noting that the SD3 narcissism scale measures primarily the grandiose aspects of this construct, while the Dirty Dozen might capture both vulnerable and grandiose features of narcissism (Maples et al., 2014), suggesting that exploring this correlation for both types of narcissism could benefit future research (Table 5).

**Table 5:** Subgroup analyses of narcissism tool.

Group	Sample size	Number Studies	Point estimate	Lower limit	Upper limit	*Z*-value	*P*-value
DDTD	348	2	−0.055	−0.159	0.051	−1.014	0.310
NARQ	297	1	0.150	0.037	0.259	2.592	0.010
NPI	785	2	0.417	0.237	0.569	4.305	0.000
SD3	11,245	22	0.350	0.313	0.386	17.054	0.000

### 4.2 Machiavellianism and mental toughness

In contrast, Machiavellianism showed no significant association with mental toughness, contradicting our second hypothesis. We initially predicted a negative correlation, hypothesizing that individuals with high Machiavellian tendencies would employ more advantageous strategies in challenging environments rather than perseverance (Birkás et al., 2015; Kilduff and Galinsky, 2017; Carré et al., 2020). Although two studies supported this hypothesis, the overall results did not, as 10 out of 13 samples did not find a significant relationship. Notably, one study using the MACH-IV scale found a moderate-to-high positive correlation between Machiavellianism and mental toughness, suggesting that mentally tough individuals feel committed to their aims and goals, similar to the dedication to personal goals characteristic of those scoring high on Machiavellianism (Sabouri et al., 2016). While this could be a sample bias, it remains significant, indicating limitations in the measures used to assess Machiavellianism.

Both the DDTD and SD3 are abbreviated versions of the MACH-IV scale (Jonason and Webster, 2010; Jones and Paulhus, 2014), implying that they might have excluded certain Machiavellian traits that more accurately reflect dark aspects rather than adaptive aspects. Furthermore, the DDTD and SD3 scales have different emphases when measuring Machiavellianism. For instance, the DDTD scale captures only interpersonal manipulation, while the SD3 provides a broader assessment, including aspects like pragmatism and cynicism (Dinić et al., 2018). Future research could compare different Machiavellian scales to further explore elements that may correlate with mental toughness.

### 4.3 Psychopathy and mental toughness

The findings on the relationship between psychopathy and mental toughness were mixed and ultimately did not support our third hypothesis, which predicted a significant negative correlation. While the majority of studies did not find a significant relationship, three studies identified a negative correlation, suggesting that individuals with higher levels of psychopathy might struggle to persistently pursue goals due to impulsive behavior (Papageorgiou et al., 2017; Vaughan et al., 2018; Zamani Sani et al., 2022). Conversely, two studies suggested a positive correlation, positing that individuals with high psychopathy scores might pursue their goals with tenacity, similar to those with strong mental toughness, by disregarding the needs of others (Sabouri et al., 2016; Szabó et al., 2022). However, the overall analysis indicated no significant correlation between psychopathy and mental toughness. This lack of significant findings may be attributed to the limited number of studies or the inadequacies of current psychopathy scales to effectively capture the multifaceted nature of psychopathy, specifically differentiating between its potentially ‘bright’ and ‘dark’ aspects. Future research should consider exploring these dimensions within psychopathy to provide a more nuanced understanding of how it relates to mental toughness.

### 4.4 Theoretical and practical implications

This meta-analysis substantively augments existing research exploring the nexus between personality traits and mental toughness, affirming that individuals with Dark Triad traits exhibit varying degrees of mental toughness, which is positively correlated only with narcissism and is unrelated to Machiavellianism and psychopathy. Our study not only synthesizes a comprehensive systematic review and meta-analysis of all pertinent prior research, but also deepens the understanding of subjective mental toughness within the Dark Triad framework, highlighting extensive opportunities for future inquiries in this domain.

Prior research has elucidated that Machiavellianism and psychopathy together form what is referred to as the Dark Dyad, distinct from narcissism, which aligns more closely yet separately with this dyad (Papageorgiou et al., 2019b; Rogoza and Cieciuch, 2020). Our findings further delineate these differences, revealing a strong association between narcissism and mental toughness, a correlation not observed with Machiavellianism and psychopathy. This differentiation underscores the critical need to examine the three domains of the Dark Triad as distinct yet interrelated constructs, each contributing uniquely to the psychological landscape (Maples et al., 2014; Dinić et al., 2018).

Beyond its theoretical implications, the positive correlation between narcissism and mental toughness corresponds with the emerging discourse on the “bright side” of narcissism, which is perceived as the most benign element of the Dark Triad, endowed with more adaptive qualities (Rauthmann and Kolar, 2012; Kaufman et al., 2019). This aspect of narcissism is positive associated with physical health (Sedikides et al., 2004), cognitive empathy (Duradoni et al., 2023) and happiness (Zheng and MacCann, 2023). At its core, narcissism involves a profound level of self-enhancement, where individuals maintain an inflated self-view that not only boosts their self-esteem but also propels them toward ambitious achievements (Morf and Rhodewalt, 1993; Sedikides, 2021). This trait has significant implications for leadership, with narcissistic leaders often driving better organizational performance through innovative strategies (Kraft, 2022). The underlying motivation for narcissists’ behaviors stems from their need to manage a fragile self-esteem, necessitating continual affirmation (Zeigler-Hill, 2006). As such, narcissists engage in immediate gratification behaviors, typically overlooking long-term repercussions (Morf and Rhodewalt, 2001). Yet, when successful, narcissists demonstrate a high degree of proactivity, unwavering belief in their capabilities, and a relentless pursuit of their objectives, epitomizing psychological toughness (Andreassen et al., 2012; Paleczek et al., 2018; Prusik and Szulawski, 2019). In particular, subgroup analyses reveal that the strongest link between narcissism and mental toughness manifests in leadership roles, underscoring the importance of this trait in organizational settings.

However, it is crucial to acknowledge that our analysis predominantly focused on grandiose narcissism. Research on the interactions between vulnerable narcissism and mental toughness remains scant, highlighting a significant gap that future studies need to address.

### 4.5 Limitations and future directions

This meta-analysis underscores several limitations inherent in the current body of research on the Dark Triad and mental toughness, notably concerning the methodologies employed. The reliance on self-report questionnaires for assessing mental toughness may introduce self-presentation or self-evaluation biases, particularly among individuals with high narcissistic traits who may overestimate their capabilities (Gabriel et al., 1994). To mitigate these biases, future research should consider alternative methods such as the Experience Sampling Method (ESM), which can reduce self-presentation biases significantly (Csikszentmihalyi and Larson, 1987), or utilize informant ratings to provide less biased reports of traits and experiences.

Moreover, the scope of research examining the intricate relationship between the Dark Triad traits and mental toughness is notably limited. This review reveals a particular scarcity of studies focusing on Machiavellianism and psychopathy. The existing studies predominantly assess these traits at a broad domain level, which constrains the depth of analysis into more nuanced relationships that might vary significantly across different subtypes or facets of these traits. Our findings indicate that while the significant positive correlation between narcissism and mental toughness may reflect the explicit inclusion of grandiose narcissism in common scales, the near-zero associations observed with Machiavellianism and psychopathy could stem from the limitations of the DDTD and SD3 scales. These scales may not adequately differentiate the finer dimensions of these traits.

Given these considerations, future research should employ more granular assessments that detail the relationships between each Dark Triad domain and mental toughness, focusing on specific aspects or subtypes to enhance understanding of these complex associations.

Additionally, gender has emerged as a significant moderating variable; however, our analysis was constrained by the mixed-gender composition of the sample pools, with many studies failing to report separate data for males and females. Future studies should aim to report correlation coefficients separately by gender to allow for more precise subgroup analyses and enhance the generalizability of the findings.

## 5 Conclusion

This systematic review and meta-analysis constitute the first exhaustive assessment of the interrelations between Dark Triad traits and mental toughness. Our findings reveal a significant association between narcissism and mental toughness. In contrast, no substantial evidence was found to support a similar link with Machiavellianism or psychopathy. This study critically consolidates existing literature while elucidating the intricate dynamics between personality traits and mental toughness.

However, the breadth of our insights is limited by various factors, including the sample sizes and the inability of the scales used to distinctly capture sub-dimensions of the Dark Triad traits. Given these limitations, it is imperative for future research to refine these methodological aspects. We advocate for more nuanced investigations that could enhance our understanding of how distinct facets of the Dark Triad correlate with mental toughness, potentially guiding targeted interventions in personality psychology.

## Data availability statement

The datasets presented in this study can be found in online repositories. The names of the repository/repositories and accession number(s) can be found in the article/supplementary material.

## Author contributions

TL: Conceptualization, Data curation, Formal analysis, Investigation, Methodology, Project administration, Resources, Software, Supervision, Validation, Visualization, Writing – original draft, Writing – review & editing. XW: Funding acquisition, Project administration, Writing – review & editing. SN: Project administration, Supervision, Writing – original draft. XX: Writing – review & editing, Conceptualization, Data curation, Formal analysis. ZN: Project administration, Supervision, Investigation, Visualization, Writing – original draft, Writing – review & editing, Conceptualization, Formal analysis, Methodology, Resources, Validation.
